# Beyond the Echo: Is Comprehensive Vascular Exploration Valuable in Cases of Non-Syndromic Thoracic Aortic Aneurysms or Bicuspid Aortic Valve?

**DOI:** 10.3390/jcdd12050167

**Published:** 2025-04-24

**Authors:** Austin Saugstad, Srekar Ravi, George Bcharah, Christine E. Firth, Hend Bcharah, Hussein Abdul Nabi, Hoang Nhat Pham, Ramzi Ibrahim, Sant J. Kumar, Mahmoud Abdelnabi, Linnea M. Baudhuin, Yuxiang Wang, Mayowa A. Osundiji, Fadi Shamoun

**Affiliations:** 1 Department of Cardiovascular Disease, Mayo Clinic, Phoenix, AZ 85054, USA; austinsaugstad@creighton.edu (A.S.); ravi.srekar@mayo.edu (S.R.); christine.firth@commonspirit.org (C.E.F.); bcharah.hend@mayo.edu (H.B.); abdulnabi.hussein@mayo.edu (H.A.N.); npham917@arizona.edu (H.N.P.); ibrahim.ramzi@mayo.edu (R.I.); sant.j.kumar@gmail.com (S.J.K.); abdelnabi.mahmoud@mayo.edu (M.A.); baudhuin.linnea@mayo.edu (L.M.B.); wang.yuxiang@mayo.edu (Y.W.); osundiji.mayowa@mayo.edu (M.A.O.); 2 Department of Cardiovascular Medicine, Creighton University, Phoenix, AZ 85012, USA; 3 Department of Laboratory Medicine and Pathology, Mayo Clinic, Rochester, MN 55905, USA; 4 Department of Clinical Genomics, Mayo Clinic, Rochester, MN 55905, USA; 5 Department of Clinical Genomics, Mayo Clinic, Phoenix, AZ 85054, USA

**Keywords:** thoracic aortic aneurysm, dissection, bicuspid aortic valve, arteriopathy, screening

## Abstract

Bicuspid aortic valve (BAV) and thoracic aortic aneurysms and dissections (TAAD) are recognized in syndromic connective tissue diseases (CTD), but most cases occur sporadically. The extent to which non-syndromic BAV or TAAD predisposes to additional arteriopathies, particularly in younger individuals, remains unclear. We retrospectively analyzed 1438 patients (mean age = 48.0, 67.7% female), excluding those with CTDs. Participants were ≤60 years old and categorized by the presence of BAV and/or TAAD. We examined co-existing arterial pathologies, including fibromuscular dysplasia, spontaneous coronary artery dissection, abdominal aortic aneurysms (AAA), mesenteric, peripheral extremity, and carotid/cerebral arteriopathies. Overall, 44.6% had either BAV or TAAD, and 27.2% had multiple arteriopathies. While vascular diseases were frequently noted, odds ratios demonstrated no significantly increased risk of extra-aortic arteriopathies in the BAV or TAAD cohorts. AAA exhibited a non-significant trend toward higher prevalence in TAAD patients. These findings support current guidelines recommending targeted imaging (transthoracic echocardiography of the aortic root and ascending aorta) over comprehensive “head-to-pelvis” screening for non-syndromic BAV or TAAD patients without additional risk factors. Ongoing genetic analyses may elucidate whether particular variants predispose to multi-site aneurysms or dissections. Consequently, targeted surveillance remains appropriate, with broader imaging reserved for patients with genetic or clinical indicators of higher risk.

## 1. Introduction

Thoracic aortic disease, including aneurysms and dissections (TAAD), leads to significant morbidity and mortality, contributing to 17,000 deaths annually in the United States [1]. Genetic influences play a prominent role in TAAD, with up to 20% of affected individuals having a first-degree relative with a dilated thoracic aorta [2,3]. Some forms of heritable TAAD exhibit multisystem features consistent with an underlying connective tissue disorder (CTD), classifying them as syndromic TAAD [4]. In cases of heritable TAAD, the predominant inheritance pattern is autosomal dominant, with varying degrees of penetrance and expressivity [3].

TAAD is commonly associated with concomitant pathology of the aortic valve. For example, bicuspid aortic valve (BAV) is a common congenital anomaly that affects 0.5% to 2.0% of adults, with a 3:1 male-to-female predominance. Patients with BAV may develop isolated aortic valve disease or aortic aneurysms, with aneurysms reported in 20% to 40% of cases [1]. Echocardiography studies indicate that over half of young individuals with normally functioning BAVs exhibit aortic dilation [5].

The prototypical CTD associated with both TAAD and BAV is Marfan syndrome, which is diagnosed based on the revised Ghent classification of 2010 and emphasizes the presence of aortic root aneurysm and ectopia lentis [6]. However, aortic dilation is also recognized in other CTDs, such as Loeys-Dietz syndrome and Ehlers-Danlos syndrome. CTDs are associated with ascending aortic and systemic arterial pathologies with a risk of spontaneous dissection even without significant dilation [7]. These examples underscore the potential benefit of thorough vascular assessments in preventing catastrophic outcomes in cases of syndromic TAAD. Current guidelines recommend comprehensive vascular imaging, including CT or MR angiography from head to pelvis, for patients with Loeys-Dietz syndrome and vascular Ehlers-Danlos syndrome [8]. For Marfan syndrome, the guidelines suggest initial screening focused on the ascending and descending aorta [8]. The necessity and frequency of comprehensive arterial assessments in non-syndromic TAAD or isolated BAV without an overt CTD are not clearly defined.

This study seeks to address the gap in knowledge regarding the prevalence and clinical significance of coexisting arteriopathies in patients with non-syndromic TAAD or BAV. By evaluating the presence of additional vascular abnormalities in these patients, we aim to assess if comprehensive vascular evaluations may be warranted.

## 2. Materials and Methods

A retrospective cohort analysis was conducted by creating a database using the Mayo Clinic systems. The database was developed from Mayo Clinic locations (Rochester, Scottsdale, and Jacksonville) from 2018–2024. Patients 18 to 60 years old with an ICD-9/10 diagnosis of arteriopathy within the database were included in this study. This relatively young cohort was chosen to examine arteriopathies less likely related to age-associated degenerative processes such as atherosclerosis or long-term hypertension. Patients with syndromic arteriopathies, including Marfan syndrome, Loeys-Dietz syndrome, and Ehlers-Danlos syndrome, were identified using specific diagnosis codes and were excluded. Those with TAAD, BAV, and other non-heritable arteriopathies including fibromuscular dysplasia (FMD), spontaneous coronary artery dissection (SCAD), carotid or cerebral artery arteriopathy, abdominal aortic arteriopathy (AAA), mesenteric artery arteriopathy, and peripheral extremity arteriopathy were identified based on ICD-9/10 codes (Appendix A Table A1). We manually reviewed the entire cohort’s inpatient and outpatient medical records in the Mayo Clinic system, including all imaging reports and notes to validate the accuracy of arteriopathy diagnoses and associated ICD-9/10 codes. A detailed chart review was also conducted for a randomly selected 10% of the included patients to ensure that the cohort was accurately representative of non-syndromic TAAD and BAV populations. Among patients with TAAD, we included patients with thoracic aortic aneurysm with or without dissection. In our study, arteriopathy encompassed non-inflammatory and non-atherosclerotic arterial diseases, specifically focusing on both aneurysms and dissections. This broad definition allowed us to include a range of vascular abnormalities beyond typical atherosclerotic arterial disease.

**Figure 1:**
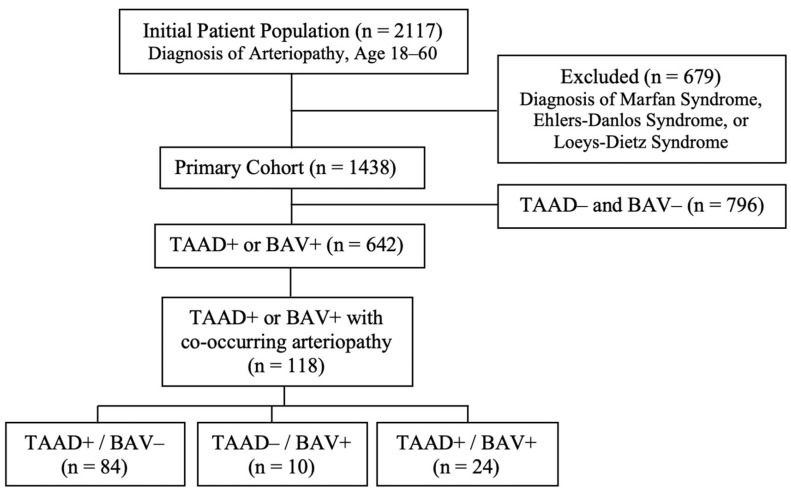
Study Population and Cohort Formation: Flowchart of patient population, exclusion criteria, and cohort design (TAAD = thoracic aortic aneurysm and dissection, BAV = bicuspid aortic valve). The final layer of the chart highlights the distribution of patients within the three sub-groups assessed in the study.

We organized the primary cohort based on the presence of TAAD and/or BAV along with other concurrent arteriopathies. We also stratified the results into subgroups (TAAD+/BAV+, TAAD+/BAV−, and TAAD−/BAV+), isolating cases of BAV without TAAD and cases of TAAD without BAV (Figure 1). Patients were grouped according to arteriopathy type, and odds ratios (OR) were calculated using Fisher’s exact test due to the expected small size of some groups. Statistical significance was set at a *p*-value of <0.05. We utilized Python’s NumPy (version 2.0.0) and SciPy (version 1.14.0) libraries for data organization and statistical analysis.

## 3. Results

Our cohort of patients with non-syndromic arteriopathies included a total of 1438 patients (Figure 1). Baseline demographic characteristics of the primary cohort (Table 1) included an average age of 48.0 years, with females constituting 67.66% of the group. Stratification of TAAD and BAV patients highlighted a male predominance (53.35%) and greater proportion of hypertension history (50.12%) in TAAD diagnosis patients, while both diagnoses comprised a greater percentage with family history of arteriopathy and co-occurring arteriopathy than the primary cohort. Table 2 presents the 1908 collective diagnoses and the prevalence of each vascular condition within the study cohort, with carotid or cerebral arteriopathy (*n* = 479) being the most common diagnosis. Notably, 44.64% (*n* = 642) of the cohort had a diagnosis of TAAD and/or BAV, of which 118 patients had an additional arteriopathy. Furthermore, 27.19% of the entire cohort (*n* = 391) had two or more arteriopathy diagnoses, as detailed in Table 3.

Our analysis (Table 4) focused on the likelihood of concurrent arteriopathies alongside TAAD or BAV. Carotid or cerebral arteriopathy was prevalent in 33.31% of the primary cohort, with an OR of concomitant TAAD or BAV of 0.03, 95% CI (0.02–0.04), *p* < 0.01. FMD affected 12.8% of the primary cohort and had an OR of 0.08, 95% CI (0.05–0.14), *p* < 0.01. SCAD was found in 11.96% of the patients, with an OR of 0.12, 95% CI (0.07–0.20), *p* < 0.01. AAA had a prevalence of 3.27%, an OR of 1.31, 95% CI (0.73–2.34), and was not statistically significant (*p* = 0.37). Mesenteric arteriopathy occurred in 13.42% of the primary cohort, resulting in an OR of 0.13, 95% CI (0.08–0.21), *p* < 0.01. Finally, peripheral extremity arteriopathy was observed in 3.40% of individuals, with an OR of 0.59, 95% CI (0.32–1.08), *p* = 0.09.

As seen in Table 4, we also analyzed the OR of our cohort for patients with both TAAD and BAV being diagnosed with another arteriopathy (*n* = 24), producing similar results. OR for additional diagnosis of FMD was 0.09, 95% CI (0.02–0.36), *p* < 0.01. OR for SCAD was 0.05, 95% CI (0.01–0.34), *p* < 0.01. Mesenteric artery arteriopathy had a corresponding OR of 0.26, 95% CI (0.11–0.6), *p* < 0.01. Carotid or cerebral arteriopathy presented an OR of 0.06, 95% CI (0.03–0.16), *p* < 0.01. Both AAA and peripheral extremity arteriopathy produced non-significant results, with OR of 1.92, 95% CI (0.89–4.2), *p* = 0.1 and OR of 0.38, 95% CI (0.09–1.58), *p* = 0.18 respectively.

We also created subgroups to assess ORs of arteriopathy in patients with independently occurring TAAD or BAV. The OR were calculated for patients with TAAD without BAV. FMD, SCAD, mesenteric artery arteriopathy, and carotid and cerebral arteriopathy yielded similar results, each with OR less than 0.25, 95% confidence intervals less than 1, and with significant *p*-values (Table 4). Notably, both AAA and peripheral extremity arteriopathy had OR greater than 1, though not reaching significance, with OR of 1.83, 95% CI (0.94–3.55), *p* = 0.08, and OR 1.17, 95% CI (0.62–2.22), *p* = 0.6 respectively.

Table 4 also shows the results of our isolated BAV subgroup. FMD, SCAD, mesenteric artery arteriopathy, and carotid and cerebral arteriopathy all had OR less than 1, with 95% confidence intervals excluding 1, and significant *p*-values. There were no co-occurring cases of peripheral extremity arteriopathy. AAA yielded an OR of 0.16, 95% CI (0.02–1.2), *p* = 0.08.

Lastly our team conducted a subsequent OR analysis of our TAAD+ and/or BAV+ patients (*n* = 642) regarding their family history and risk of co-arteriopathy, resulting in an OR 0.9, 95% CI (0.49–1.74), though not reaching significance, with a *p*-value of 0.8.

## 4. Discussion

Our cross-sectional analysis assessed vascular involvement beyond the thoracic aorta in a cohort of non-syndromic patients with BAV and TAAD. Patients less than 60 years old were included to minimize the influence of atherosclerotic changes, which are more common in older populations, and to isolate intrinsic vascular pathologies. By excluding age-related degenerative processes and known systemic connective tissue disorders, we concentrated on a demographic where screening guidelines remain less defined and the risk of further arteriopathy is unclear. There are significant financial consequences when considering screening for systemic arteriopathy in patients with BAV and TAAD, thus warranting an evaluation of the frequency of these co-occurring pathologies. TAAD and BAV are not uncommon, with BAV present in nearly 1–2% of the population [9], while TAAD affects an estimated 7.6 per 100,000 individuals [10], highlighting the impact of this study.

According to current ACC and AHA guidelines, the recommended screening protocol for sporadically identified TAAD in the absence of syndromic features includes surveillance transthoracic echocardiography (TTE) imaging every 6 to 12 months to monitor the rate of aortic enlargement. Depending on the rate of dilation, subsequent screenings should occur every 6 to 24 months. For patients with BAV, an assessment of the aortic root and ascending aorta is recommended (CT or MRI if not visualized by TTE). Additionally, it is advised to obtain a multigenerational family history for all patients with aortic root and ascending aortic disease, and genetic testing is indicated if a positive family history or other risk factors are present (such as syndromic physical features). For patients with BAV and TAAD or dissections, screening of first-degree relatives is recommended to identify similar, potentially unrecognized, asymptomatic conditions. Comprehensive vascular imaging (head to pelvis) is recommended for syndromic patients with Loeys-Dietz syndrome and vascular Ehlers-Danlos syndrome (EDS), while patients with Marfan syndrome typically receive imaging of the ascending and descending aorta (chest to pelvis) [8].

For patients with BAV, an assessment of the aortic root and ascending aorta is recommended (CT or MRI if not visualized by TTE). Those with syndromic physical features should undergo genetic testing. If BAV with ascending aortic disease is identified, TTE screening of all first-degree relatives is recommended [11,12,13]. Recommendations for patients with Marfan and Loeys-Dietz syndrome include aortic imaging at initial diagnosis and six months thereafter to establish if enlargement is occurring, with subsequent annual screening [8]. There is also a Class 1 recommendation for patients with Loeys-Dietz syndrome to complete a baseline CT or MRI from head to pelvis to evaluate for systemic arteriopathy. Though surveillance screening recommendations for vascular Ehlers-Danlos syndrome are not yet defined, general protocols are similar and include baseline head-to-pelvis CT or MRI imaging, with annual follow-up if abnormalities are observed, and every two years if stable [8].

For patients with non-syndromic aortic disease such as BAV and TAA or dissections, the risk of developing widespread systemic arteriopathies and the need for additional screening beyond the initial TTE remains uncertain. Various genes have been implicated in TAAD, and genetic testing can identify pathogenic mutations that significantly increase a patient’s risk for aneurysm or dissection at other sites. Investigative screening for other systemic arteriopathies in non-syndromic patients is dependent upon the specific genetic variants identified (ACTA2, MYH11, PRKG1, MYLK, and LOX) during genetic screening, though no recommendation for complete vascular investigation is provided for those without an identified mutation [14,15]. As genetic testing becomes more prevalent, the identification of additional genes associated with BAV and TAAD will aid in the earlier diagnosis of asymptomatic non-syndromic cases and help risk-stratify who should receive more comprehensive vascular imaging [16].

Our study evaluated the risk of additional arteriopathies in this cohort by analyzing the prevalence and odds ratios of co-existing pathologies in other vascular beds. We conducted a detailed assessment of co-existing arteriopathies across our primary and secondary cohorts. The analysis revealed significantly decreased associations with concurrent diagnoses of FMD, SCAD, mesenteric artery arteriopathy, peripheral extremity aneurysms, and carotid or cerebral arteriopathy across all cohorts. There was a non-significant increased association with AAA in the TAAD+ subgroups. Therefore, while the lack of significance among TAAD/BAV patients with abdominal aortic aneurysms may not warrant routine screening, this does represent a potential area for further research. Our study showed no significantly increased associations between TAAD or BAV and other arteriopathies to support imaging of other vascular beds in this population. Future research will assess the genetic profile of our patients to see if those who developed systemic disease had an underlying genetic abnormality.

### Limitations and Future Directions

Prospective scans were not performed on the included patients, nor was comprehensive scanning completed on all patients, which may result in missed cases of arteriopathy. As a cross-sectional study, we cannot establish causality or monitor the progression of vascular conditions over extended periods. While attempting to control for atherosclerotic disease with the study cohort, underlying atherosclerosis cannot be ruled out as a contributing factor to co-existing arteriopathy. Additionally, patient demographic data were obtained at the time of study, rather than at time of diagnosis. Thus, our ability to further investigate key differences in initial clinical presentation was limited. The use of ICD-9/10 coding to define diagnoses introduced potential variability in the accuracy of diagnoses, although validation was done as described in the methods.

## 5. Conclusions

We evaluated for additional arteriopathies in patients with TAAD or BAV that did not have a vascular syndrome or suspected disease due to atherosclerosis. The results showed negative associations or non-significant associations between TAAD and/or BAV and other studied arteriopathies. Our findings support current clinical guidelines, which do not recommend comprehensive vascular imaging for non-syndromic cases of TAAD or BAV patients without additional risk factors. However, our results indicate a non-trivial prevalence of other arteriopathies in this population, as well as a non-significant correlation between TAAD and AAA, which may warrant further study.

## Figures and Tables

**Table 1:** Primary cohort demographic and clinical characteristics ^a^.

Characteristic	Primary Cohort (*n* = 1438)	TAAD (*n* = 433)	BAV (*n* = 351)
Median Age (years)	48.0 (+/−9.36)	49.71 (+/−8.88)	46.68 (+/−10.13)
Female sex (*n*, %)	973 (67.66)	202 (46.65)	190 (54.13)
History of Hypertension (*n*, %)	560 (38.94)	217 (50.12)	129 (36.75)
Smoking History (*n*, %)			
Current	92 (6.4)	31 (7.16)	27 (7.69)
Former	343 (23.85)	88 (20.32)	68 (19.37)
Never	962 (66.9)	310 (71.59)	265 (75.5)
Family History of Arteriopathy (*n*, %)	121 (8.41)	59 (13.63)	37 (10.54)
Co-occurring arteriopathy (*n*, %) *	391 (27.19)	198 (45.72)	150 (42.74)

^a^ Plus-minus values indicate mean +/− standard deviation. * Indicates patients with at least two different diagnoses of arteriopathy.

**Table 2:** Prevalence of Each Arteriopathy Diagnosis in the Primary Cohort.

Diagnosis	Prevalence
Thoracic Aortic Aneurysm and Dissection	433
Bicuspid Aortic Valve	351
Carotid or Cerebral Arteriopathy	479
Mesenteric (Celiac, SMA, Renal) Arteriopathy	193
Abdominal Aortic Arteriopathy	47
Peripheral Extremity Arteriopathy	49
Fibromuscular Dysplasia	184
Spontaneous Coronary Artery Dissection	172
Total	1908

**Table 3:** Diagnosis Stratification Among the Primary Cohort.

Diagnosis/Arteriopathy	*n* (%)
Patients with 2 or more diagnosis	391 (27.19)
TAAD or BAV with other arteriopathy	118 (8.21)
Both TAAD + BAV with other arteriopathy	24 (1.67)
Both TAAD + BAV without other arteriopathy	124 (8.62)

**Table 4:** Subgroup Analysis of Co-occurring Arteriopathy among Patients with BAV and TAAD.

Co-Existing Vascular Condition	Patient Group	#	OR	95% CI Lower	95% CI Higher	*p*-Value
Fibromuscular Dysplasia (FMD)	TAAD and/or BAV	14	0.08	0.05	0.14	<0.01
	TAAD+/BAV+	2	0.09	0.02	0.36	<0.01
	TAAD+/BAV−	8	0.10	0.05	0.21	<0.01
	TAAD−/BAV+	4	0.07	0.03	0.20	<0.01
Spontaneous Coronary Artery Dissection (SCAD)	TAAD and/or BAV	18	0.12	0.07	0.20	<0.01
	TAAD+/BAV+	1	0.05	0.01	0.34	<0.01
	TAAD+/BAV−	16	0.24	0.14	0.41	<0.01
	TAAD−/BAV+	1	0.02	<0.01	0.14	<0.01
Abdominal Aortic Arteriopathy (AAA)	TAAD and/or BAV	24	1.31	0.73	2.33	0.37
	TAAD+/BAV+	8	1.92	0.88	4.20	0.10
	TAAD+/BAV−	15	1.83	0.94	3.55	0.08
	TAAD−/BAV+	1	0.16	0.02	1.20	0.08
Mesenteric Arteriopathy	TAAD and/or BAV	22	0.13	0.08	0.21	<0.01
	TAAD+/BAV+	6	0.26	0.11	0.60	<0.01
	TAAD+/BAV−	15	0.20	0.12	0.34	<0.01
	TAAD−/BAV+	1	0.02	0.00	0.13	<0.01
Peripheral Extremity Arteriopathy	TAAD and/or BAV	16	0.59	0.32	1.08	0.09
	TAAD+/BAV+	2	0.38	0.09	1.58	0.18
	TAAD+/BAV−	14	1.17	0.62	2.22	0.63
	TAAD−/BAV+	0	-	-	-	-
Carotid or Cerebral Arteriopathy	TAAD and/or BAV	24	0.03	0.02	0.04	<0.01
	TAAD+/BAV+	5	0.06	0.03	0.16	<0.01
	TAAD+/BAV−	16	0.04	0.03	0.07	<0.01
	TAAD−/BAV+	3	0.01	<0.01	0.03	<0.01

## Data Availability

The data supporting the findings of this study are not publicly available due to privacy and ethical restrictions.

## Abbreviations

The following abbreviations are used in this manuscript:

AAA	Abdominal Aortic Aneurysm
ACC	American College of Cardiology
AHA	American Heart Association
BAV	Bicuspid Aortic Valve
CI	Confidence Interval
CT	Computed Tomography
CTD	Connective Tissue Disorder
EDS	Ehlers-Danlos Syndrome
FMD	Fibromuscular Dysplasia
ICD	International Classification of Diseases
MRI	Magnetic Resonance Imaging
OR	Odds Ratio
SCAD	Spontaneous Coronary Artery Dissection
SMA	Superior Mesenteric Artery
TAAD	Thoracic Aortic Aneurysm and Dissection
TTE	Transthoracic Echocardiography

## Appendix A

**Table A1:** List of ICD 9/10 codes used to identify patients for cohort enrollment.

Vascular Condition	ICD Codes
Fibromuscular Dysplasia (FMD)	447.3, 447.8, I77.3
Spontaneous Coronary Artery Dissection (SCAD)	414.11, 414.12, 414.19, 417.1, 747.3, 747.6, I25.4, I25.41, I25.42, I28.1
Bicuspid Aortic Valve (BAV)	746.4, Q23.1
Marfan’s Syndrome	759.82, Q87.4, Q87.40, Q87.41, Q87.410, Q87.418, Q87.42, Q87.43
Thoracic aortic arteriopathy	441, 441.01, 441.1, 441.2, 441.5, 441.6, 441.7, 441.9, 747.29, I71, I71.0, I71.00, I71.01, I71.03, I71.1, I71.2, I71.5, I71.6, I71.8, I71.9, I79.0, Q25.43, Q25.49
Abdominal aortic aneurysm/dissection	V17.49, 441.02, 441.03, 441.3, 441.4, I71.02, I71.3, I71.4
Mesenteric (celiac, SMA, renal) arteriopathy	442.1, 442.83, 442.84, 442.89, 443.22, 443.23, 443.29, I72.2, I72.8, I72.9, I77.7, I77.70, I77.73, I77.79
Peripheral extremity aneurysm/dissection	442, 442.2, 442.3, 442.82, I72.1, I72.4, I77.72, I77.76, I77.77, I72.3
Carotid or cerebral arteriopathy	430, 437.3, 442.81, 443.21, 443.24, I60.7, I60.9, I67.0, I67.1, I72.0, I72.5, I72.6, I77.71, I77.74, I77.75
Ehler’s Danlos	756.83, Q79.6, Q79.60, Q79.61, Q79.62, Q79.63, Q79.69
Loeys-Dietz Syndrome	Q87.89
